# Pure Red Cell Aplasia and Lymphoproliferative Disorders: An Infrequent Association

**DOI:** 10.1100/2012/475313

**Published:** 2012-04-19

**Authors:** Efthymia Vlachaki, Michael D. Diamantidis, Philippos Klonizakis, Styliani Haralambidou-Vranitsa, Elizabeth Ioannidou-Papagiannaki, Ioannis Klonizakis

**Affiliations:** Department of Haematology, Second Department of Internal Medicine, Aristotle University of Thessaloniki, Hippokration General Hospital, 546 42, Thessaloniki, Greece

## Abstract

Pure red cell aplasia (PRCA) is a rare bone marrow failure syndrome defined by a progressive normocytic anaemia and reticulocytopenia without leukocytopenia and thrombocytopenia. Secondary PRCA can be associated with various haematological disorders, such as chronic lymphocytic leukaemia (CLL) or non-Hodgkin lymphoma (NHL). The aim of the present review is to investigate the infrequent association between PRCA and lymphoproliferative disorders. PRCA might precede the appearance of lymphoma, may present simultaneously with the lymphoid neoplastic disease, or might appear following the lymphomatic disorder. Possible pathophysiological molecular mechanisms to explain the rare association between PRCA and lymphoproliferative disorders are reported. Most cases of PRCA are presumed to be autoimmune mediated by antibodies against either erythroblasts or erythropoietin, by T-cells secreting factors selectively inhibiting erythroid colonies in the bone marrow or by NK cells directly lysing erythroblasts. Finally, focus is given to the therapeutical approach, as several treatment regimens have failed for PRCA. Immunosuppressive therapy and/or chemotherapy are effective for improving anaemia in the majority of patients with lymphoma-associated PRCA. Further investigation is required to define the pathophysiology of PRCA at a molecular level and to provide convincing evidence why it might appear as a rare complication of lymphoproliferative disorders.

## 1. Introduction

Pure red cell aplasia (PRCA), initially described by Kaznelson in 1922 [1], is a rare disorder, characterized by the presence of a severe normochromic, most frequently normocytic anaemia and reticulocytopenia (<1%) associated with a marked reduction of the bone marrow erythroid precursors (<5%) and normal production of the white blood cell and megakaryocytic lineages [2–4]. Therefore, it is presumed that the defect lies within the erythroid precursors and not within the stem cells as seen in aplastic anaemia [2].

PRCA is a rare bone marrow failure disorder without geographic or racial predilection. All ages can be affected. If present in children, it is called transient erythroblastopenia of childhood, an acute and self-limited disorder, which might be difficult to distinguish from congenital causes of anaemia, such as Diamond-Blackfan anemia. Former nosology included various terms like chronic hypoplastic anaemia, pure red-cell agenesis, primary red-cell anaemia, and erythrophthisis [2].

The classification of PRCA involves (i) the congenital disorders of PRCA, which usually manifest themselves early in life, (ii) primary PRCA, which occurs in the absence of any underlying disorder and is predominantly autoimmune in origin, despite the idiopathic cases, and (iii) the acquired or secondary PRCA syndrome. The acquired form of PRCA presents either as an acute self-limited disease, mainly encountered in children, or as a chronic illness, more commonly seen in adults. Thus, PRCA may present as a primary haematological disorder in the absence of any other disease or secondary to parvovirus infection, collagen vascular disease, leukaemia, lymphoma, other lymphoproliferative disorders, thymoma, solid tumors, treatment with recombinant human erythropoietin or other drugs, ABO-incompatible haematopoietic stem cell transplantation and pregnancy (Table 1) [5]. The course can be acute and self-limiting or chronic with rare spontaneous remissions, depending on the cause [6].

Since PRCA is a rare disorder, haematologists should be alert to include PRCA in the differential diagnosis in cases of severe normochromic and normocytic anaemia combined with reticulocytopenia and normal production of the white blood cell and megakaryocytic lineages. Clinicians often fail to diagnose PRCA. The experience of our center includes only few cases of PRCA during a period of twenty years, involving only one published case of PRCA combined with small lymphocytic lymphoma (SLL) [3].

Acquired forms of PRCA must be distinguished from congenital forms of PRCA, such as Diamond-Blackfan anaemia, Fanconi anaemia, and congenital dyserythropoietic anaemias. Acquired PRCA occurring in childhood (transient erythroblastopenia of childhood) may be difficult to distinguish from Diamond-Blackfan anaemia. A history of normal blood counts, late onset of manifestation, and transient disease course are characteristic of transient erythroblastopenia of childhood. Interestingly, a distinction between myelodysplastic syndrome (MDS) with erythroid hypoplasia and idiopathic PRCA might be more difficult [2].

The aim of the present review is to investigate the infrequent association between PRCA and lymphoproliferative disorders. Possible pathophysiological molecular mechanisms to explain the infrequent association between PRCA and lymphoproliferative disorders are provided, according to the literature. Finally, focus is given to the therapeutical approach, as several treatment regimens have failed for PRCA.

## 2. PRCA Cases Associated with Lymphoproliferative Disorders

As stated above, PRCA has rarely been reported in association with lymphoproliferative disorders, as one of the autoimmune diseases seen during the course of lymphoid malignancies. Nevertheless, the relationship between PRCA and the underlying disorders remains to be elucidated. PRCA might precede the appearance of a lymphoproliferative disorder, may present simultaneously with the lymphoid neoplastic disease, or might appear following the lymphomatic disorder, either in relapse or interestingly, even in remission of it [7]. The Japanese Collaborative Study Group for PRCA reports 8 cases with lymphoma from a cohort of 185 PRCA patients in a period of time between 1990 and 2006 in Japan. Hence, acquired PRCA, because of lymphoma, is infrequent, as it involves approximately one new case every two years in Japan [7]. A review of the literature in the same study has provided 22 additional cases of lymphoproliferative disorders-associated PRCA worldwide from 1978 to 2007 [7]. Various histologic subtypes of lymphoid malignancies in association with PRCA, either of the B-cell type or of the T-cell type, have been documented (Table 2) [3, 4, 7–14]. The prevalence of PRCA associated with lymphoproliferative disorders may be higher, as there might exist cases which have not been published.

## 3. Pathophysiological Mechanisms

### 3.1. Evidence Favoring the Notion of B-Cell Participation in the Pathogenesis of Secondary PRCA, Related to Lymphoproliferative Disorders

Early demonstrations of factors in the patients' sera that inhibit erythropoiesis *in vivo* and *in vitro* led to the hypothesis that these inhibitors could be IgG antibodies, either complement-binding, directly cytotoxic, targeting erythroblasts *in vitro*, or inhibiting haemoglobin synthesis [15]. These IgG antibodies might derive directly from a possible B-cell clone or could be polyclonal [15]. The antigenic targets for autoantibodies have not been well characterized, but various stages of erythroid differentiation can be affected, as seen in the reduction of erythroid burst-forming units or erythroid colony-forming units. In certain cases of antibody-mediated PRCA, the involvement of the complement system is prerequisite to disease causation [2]. Some other cases of adult autoimmune PRCA might be induced by antibodies produced following a viral or bacterial infection that might cross-react with erythroid precursor cells or erythropoietin [16].

In addition, a possible model for antibody-induced red cell aplasia is the identification of PRCA associated with antierythropoietin antibodies in rare cases [2]. The possible molecular mechanism of antibody-mediated reduction of erythropoiesis includes direct complement-mediated lysis of red-cell progenitors and the formation of immune complexes with erythropoietin, which result in functional inactivation and the removal of erythropoietin from the circulation [16]. An alternative notion involves obstruction of the erythropoietin receptor or another red-cell signalling pathway by anti-erythroblast antibodies [16].

### 3.2. Evidence Supporting the Idea of a T-Cell or NK-Cell Implication in the Pathogenesis of Secondary PRCA, Related to Lymphoproliferative Disorders

Initial findings suggesting the implication of T cells, either polyclonal or monoclonal, in the pathogenesis of PRCA, due to lymphoproliferative disorders, resulted from the coexistence of thymoma and PRCA, or T-LGL and PRCA [14]. Interestingly, PRCA is the second most common haematologic disease found in T-LGL patients, exceeded only by autoimmune hemolytic anaemia [14]. The mechanism of erythroid inhibition may vary and may include direct cytotoxic T-lymphocyte-mediated attack of the erythroid precursors or production of soluble inhibitory and proapoptotic cytokines by the cytotoxic T-lymphocytic population that directly affect the erythroid series [2].

Interestingly, LGL expansion may be the disorder most commonly associated with PRCA. These LGLs may be of T-cell type or of natural killer (NK)-cell type. T-LGLs express CD3 and a T-cell receptor (TCR) of *αβ*- or *γδ*-type. On the contrary, NK-LGLs are CD3 negative, thus they do not express a TCR at the cell surface. Typically, these NK-LGLs do not rearrange the TCR genes *α*, *β*, *γ*, and *δ*. Clonal expansion of T-LGLs of *γδ*-type has been described in PRCA cases, combined with malignancy [17]. Hence, apart from the direct CD8(+) cytotoxic T-lymphocytic mediated attack of the erythroid precursors, related to major histocompatibility complex I (MHC-I), there is a second mechanism of T-cell toxicity, implicating a clonal expansion of T-LGLs, which is independent of MHC-I-restricted CD8(+) cytotoxic T-lymphocytes.

The malignant T-LGLs, in the latter mechanism, carry killer cell inhibitory receptors (KIRs), which inhibit the lytic machinery of the killer cell, when the target cell expresses the HLA class I antigen to which the particular KIR binds [17]. Tumor cells, frequently, lose HLA class I antigens as an escape mechanism to avoid recognition by MHC-I-restricted CD8(+) T-lymphocytes. Nevertheless, in this case, they can be destroyed by cytotoxic MHC-unrestricted killer cells (T-LGLs) expressing KIRs. Thus, when red-cell progenitors progressively lose HLA class I antigens from their surface, KIRs fail to deliver an “off” signal to the killer T-LGLs, leading to red-cell destruction by T-LGLs [17]. In the aforementioned condition, the activated killer T-LGLs, via their TCR, recognize ligands on the red-cell progenitors, thereby triggering red-cell destruction and causing PRCA. These ligands/antigens have not been well described. LGL-lymphokine secretion, causing red-cell lysis has also been proposed. Hence, in addition to T-lymphocytes expressing *αβ*- TCR, T cells with a *γδ*- TCR can mediate PRCA [2]. The myeloid cells express normal levels of HLA class I molecules that bind to the KIRs on the T-LGLs and deliver “off” signals to the killer cells. The same mechanisms exist for NK-LGLs, with the difference that NK-LGLs do not express a TCR, but may be positively triggered by circulating antibody against red-cell antigens, activating NK-receptors and adhesion molecules [17].

Finally, an interesting theory has been reported. There seems to be a balance between the various degrees of expression of HLA class I alleles on the surface of possible target cells. Thus, while one HLA class I allele may present an antigenic peptide to T-LGLs for lysis, other HLA class I alleles may at the same time inhibit cytolysis by binding to the KIRs [18].

### 3.3. PRCA Pathogenesis: B Cell or T Cell or Both? Clonal or Polyclonal?

The question of whether a B-cell clone or a T-cell clone participates in the pathogenesis of secondary PRCA, due to malignant lymphomas or lymphoproliferative disorders, is intriguing. Most cases of PRCA are presumed to be autoimmune mediated by antibodies against either erythroblasts or erythropoietin, by T-cells secreting factors selectively inhibiting erythroid colonies in the bone marrow or by natural killer (NK) cells directly lysing erythroblasts [11]. The above-mentioned pathophysiological mechanisms might coexist to the same patient. An example is the concurrent presence of T-LGL and B-NHL or T-LGL and B-CLL to patients diagnosed with PRCA [14].

An interesting mechanism has been reported concerning antibodies against red-cell progenitors binding to CD16 on the LGLs. Hence, cytotoxicity can be induced by antibody cross-linking the TCR of the T-LGLs with the Fc receptor on target cells or by antibody cross-linking the Fc receptor on the T-LGLs or NK-LGLs (CD16) with any specific ligand for the antibody on the target cells. Thus, T-LGLs (*αβ*-TCR or *γδ*-TCR) may use the same mechanism of NK-mediated lysis. Then, the TCR is not directly involved in the recognition of red-cell progenitors. The latter involves an interaction between antibodies (B cells) and T cells (LGLs or NK-LGLs) for the pathogenesis of PRCA in lymphoproliferative disorders [19, 20]. The existing molecular mechanisms, based on the literature, linking PRCA and lymphoproliferative disorders are highlighted in Table 3.

Most T-LGL proliferations associated with PRCA are probably clonal, even though polyclonal expansions of T-LGLs, such as following an immune response against an unknown antigen, may be linked to autoimmunity [19]. The molecular demonstration of clonality in some LGL patients might mean dominance of one or a few clones within a polyclonal population of LGLs. Interestingly, many patients with T-LGL expansions have a chronic course, and the demonstration of clonality by itself does not obligatory mean malignancy. Benign clonal T-cell expansions have been described following viral infections and in elderly humans [21]. Indeed, it has been reported that initial clonal T-LGL proliferations might no longer be detectable, after the development of autoimmunity [19].

Similar notions apply for NK-LGL expansions, although clonality is difficult to establish in NK-LGL proliferations, because of the lack of TCR gene rearrangements in NK-LGLs [22]. However, some of the NK-LGL lymphocytosis have been shown to be polyclonal [23]. The majority of patients with T-LGL and NK-LGL proliferations may have lower LGL expansions that only become apparent if an absolute lymphocytosis or an increased proportion of peripheral lymphocytes with an LGL phenotype is specifically investigated [16].

## 4. Treatment

The major goal in treating PRCA is to induce a remission with the recovery of erythropoiesis, thereby providing relief from transfusions and avoiding transfusion-related complications. The therapeutic strategy usually focuses on immunosuppressive treatments, until a remission is obtained. Remissions have been achieved by treatment with corticosteroids, cyclophosphamide, cyclosporine A (one of the leading drugs for PRCA treatment, a T-cell targeted therapy), antithymocyte globulin, splenectomy, and plasmapheresis. Recently, the efficacies of the anti-CD20 monoclonal antibody, rituximab, and anti-CD52 monoclonal antibody, alemtuzumab, have been reported to induce remission in resistant cases of PRCA. Other therapeutic options include intravenous immunoglobulin, thymectomy, or peptide-based agonists for the erythropoietin receptor (Table 4) [5].

Immunosuppressive therapy and/or chemotherapy (immunosuppressive properties in cases of malignant lymphoma) are effective for improving anaemia in the majority of patients with PRCA, due to lymphoproliferative disorders. However, in a significant portion of patients, remission of anaemia is maintained, even without immunosuppressive therapy. These results suggest indeed that durable maintenance-free remission of anaemia may be obtained in lymphoproliferative disorders-associated PRCA and that PRCA may occur as paraneoplastic syndrome of lymphoid malignancies [7].

The mechanism of action of rituximab in PRCA is not entirely clear. The most obvious explanation is the elimination of antibody producing tumor cells. However, the rapid and dramatic response even with the persistence of residual neoplastic cells in some patients raises the possibility of other mechanisms, as well [11]. Moreover, intravenous immunoglobulin contains neutralizing antibody against parvovirus B19 and has been reported to be effective for PRCA cases associated to parvovirus B19 infection [5].

The anti-CD52 monoclonal antibody alemtuzumab targets against CD52 antigen, present on B and T lymphocytes, NK cells, monocytes/macrophages, dendritic cells, and eosinophils, but not on human haematopoietic stem cells. The rationale behind the use of alemtuzumab in PRCA is that T lymphocytes are thought to play an important role in the autoimmune pathogenesis of PRCA, as they are involved in the control of the expansion of immunoglobulin-producing autoreactive B-lymphocyte clones [5]. Finally, PRCA due to autoantibodies against endogenous erythropoietin might occur, especially to patients previously treated with recombinant human erythropoietin. The serum of PRCA patients with anti-erythropoietin antibody inhibits the growth of erythroid progenitor cells *in vitro*. That is why peptide-based erythropoietin receptor agonists for treating PRCA have been developed [5].

## 5. Discussion: Additional Comments

It is well established that viral infections, such as parvovirus B19 [24] or Epstein-Barr Virus (EBV) [8], may cause PRCA, especially in immunocompromised patients (due to Human Immunodeficiency Virus (HIV) infection, or after chemotherapy or after transplantation). A case of chronic parvovirus B19 infection leading to red cell aplasia following treatment of Hodgkin lymphoma has been recently reported [25]. These patients might be unable to mount a neutralizing antibody response against the autoimmune background of PRCA, because of persistent bone marrow and host immunity insufficiency. However, the evaluation of EBV or parvovirus B19 infection with serological tests in immunocompromised patients with secondary PRCA, is not reliable, as antibody production is absent or minimal [24].

From the point of view regarding the pathogenesis of lymphoproliferative disorder-associated PRCA, it is interesting that positive Coombs test is often associated with PRCA, which suggests the role of autoreactive antibody in such cases [7]. However, the difference between autoimmune hemolytic anaemia (AIHA) noted in lymphomas and lymphoproliferative disorder-induced PRCA, seems to be the level of maturity of the red-cell line, which receives the immune attack. In PRCA, an autoreactive antibody against the erythroid precursors might be responsible for the disease, whereas in AIHA the autoreactive antibody targets mature erythroid cells. After an analysis of AIHA or Evans' syndrome, encountered in patients with NHL, it has been reported that warm antibody-mediated AIHA is more frequent in B-cell lymphomas, while cold antibody-mediated AIHA mainly occurs in T-cell lymphomas [26]. Additionally, in all cases of AITL associated with PRCA, positive Coombs test has been noted [12]. Coexistence of AIHA and PRCA in malignant lymphoma has also been reported [8].

In addition, the expansion of LGLs can be easily missed or underestimated in the analysis of bone marrow histology of PRCA patients, because it may be rather scanty. In some patients with documented clonal T-LGL proliferations, the clonal LGLs account for less than 5% of the bone marrow cells. It is possible that in some anaemic patients with increased levels of LGLs, not all erythroblasts are destroyed by the LGLs and the bone marrow reveals merely reduced erythropoiesis, but not a true PRCA [16]. Occasionally, clonally expanded lymphocytes with a characteristic CD3(+), CD57(+) phenotype may not have LGL morphology on a peripheral smear, but may indeed represent *in vivo *antigen-activated cytotoxic effector T cells. Thus, an increase of CD3(+)/CD56(−) or CD3(−)/CD56(+) cells by peripheral blood flow cytometry and/or an inverted CD4(+)/CD8(+) cell ratio suggests the existence of LGL leukaemia [5]. Importantly, a higher percentage of erythroid progenitors (>5%) does not exclude a diagnosis of PRCA in an appropriate clinical setting [27].

A very interesting aspect of lymphoproliferative disorder-associated PRCA is that, while after chemotherapy, the disorder might be at remission, PRCA may insist. Thus, while the number of neoplastic cells in the bone marrow might decline after chemotherapy, PRCA may continue to be present [13]. However, the accurate detection of lymphomatic bone marrow involvement in patients with PRCA is of crucial importance because of the therapeutic consequences. Bone marrow trephine biopsy (BMB) is currently regarded as the method of choice for the evaluation of the bone marrow in lymphoma, but it is invasive, has a risk of complications, and lacks sufficient sensitivity, due to the possibility of sampling errors. Bone marrow imaging, if accurate, may partially replace BMB and may improve the sensitivity of BMB by guiding the biopsy to the location that appears to be involved by lymphoma at imaging [28].

The molecular, genetic, and immunologic events implicated in the pathogenesis of lymphoproliferative disorder-associated PRCA are complex and not yet fully elucidated. An overview on speculating the most important mechanisms, based on existing literature evidence has been provided. Moreover, case reports in conjunction with different entities of B- T- NK-cell lymphoproliferative malignancies inducing PRCA have been described. Further investigation is required to define the pathophysiology of PRCA at a molecular level and to provide convincing evidence why it might emerge as a rare complication of lymphoproliferative disorders.

##  Authors' Contribution

Efthymia Vlachaki performed literature review, analyzed and interpreted data, and wrote the paper; Michael D. Diamantidis performed literature review, analyzed and interpreted data, and wrote the paper; Efthymia Vlachaki and Michael D. Diamantidis contributed equally to the paper; Philippos Klonizakis performed literature review and analyzed and interpreted data; Styliani Haralambidou-Vranitsa analyzed and interpreted data and corrected the paper; Elizabeth Ioannidou-Papagiannaki analyzed and interpreted data and corrected the paper; Ioannis Klonizakis analyzed and interpreted data and corrected the paper.

## Figures and Tables

**Table 1:** Classification of PRCA [2, 5].

Congenital PRCA	
Primary autoimmune PRCA	
Primary idiopathic PRCA	
Secondary (acquired) PRCA, due to thymoma	
Secondary PRCA, because of haematological malignancies (CLL, T-LGL/NK-LGL leukaemia, myeloma, NHL, MDS, ALL)	
Secondary PRCA, related to infections (parvovirus B19, EBV, HIV, CMV, viral hepatitis, leishmaniasis, staphylococcemia)	
Acquired PRCA, due to ABO-incompatible haematopoietic stem cell transplantation	
Acquired PRCA, related to pregnancy	
Secondary PRCA, because of autoimmune diseases (Sjögren's syndrome, SLE, RA, mixed connective tissue disease, autoimmune hepatitis)	
Secondary PRCA, because of previous treatment with recombinant human erythropoietin or other drugs (azathioprine, allopurinol, penicillin, linezolid, estrogens, ticlopidine, lamivudine, fludarabine)	
Acquired PRCA, related to solid tumors (thyroid cancer, renal cell carcinoma)	
Acquired PRCA, due to severe nutritional deficiencies	

**Table 2:** Histologic subtypes of lymphoid malignancies in association with PRCA [3, 4, 7–14]. Literature review was performed in references [4, 7, 11, 12] and the additional cases have been added to the Table.

Histologic subtype of lymphoproliferative disorder	Number of Cases (61 Total)	References
Diffuse large B-cell lymphoma (DLBCL)	8	Hirokawa et al., 6 cases, 2009 [7], Sung et al., 1 case, 2007 [8], Suzuki et al., 1 case, 2007 [9]
Hodgkin's disease	4	Hirokawa et al., 4 cases, 2009 [7]
Small lymphocytic lymphoma (SLL)	1	Vlachaki et al., 1 case, 2008 [3]
B chronic lymphocytic leukaemia (B-CLL)	7	Castelli et al., 1 case, 2002 [10] Narra et al., 6 cases, 2006 [11]
Nodal marginal zone lymphoma (NMZL)	1	Hirokawa et al., 1 case, 2009 [7],
Splenic marginal zone lymphoma (SMZL)	3	Narra et al., 1 case, 2006 [11], Anastasiadis et al., 2 cases, 2009 [4]
Mycosis fungoides	1	Hirokawa et al., 1 case, 2009 [7]
Angioimmunoblastic T-cell lymphoma (AITL)	12	Hirokawa et al., 4 cases, 2009 [7], Choi et al., 8 cases, 2010 [12]
Follicular lymphoma (FL)	7	Hirokawa et al., 6 cases, 2009 [7], Kuramoto et al., 1 case, 1998 [13]
Precursor T-lymphoblastic lymphoma (T-LBL)	1	Hirokawa et al., 1 case, 2009 [7]
Adult T-cell leukaemia/lymphoma (ATLL)	1	Hirokawa et al., 1 case, 2009 [7]
T-cell large granular lymphocyte (T-LGL) leukaemia	15	Go et al., 15 cases, 2001 [14]

**Table 3:** Proposed molecular mechanisms for PRCA, due to lymphoproliferative disorders [2, 15–17, 19, 20].

*B cells*	
IgG antibodies, either complement-binding, directly cytotoxic, targeting erythroblasts *in vitro* or inhibiting haemoglobin synthesis [15].	
Antibodies produced following a viral or bacterial infection that might cross-react with erythroid precursor cells or erythropoietin [16].	
Anti-erythropoietin antibodies, immune complexes with erythropoietin, which result in functional inactivation [2, 16].	
Obstruction of the erythropoietin receptor by anti-erythroblast antibodies [16].	

*T cells, NK cells*	
MHC-I-restricted CD8(+) cytotoxic T-lymphocytes [17].	
MHC-unrestricted CD3(+) T-LGLs with TCR expressing KIRs [17].	

*B cells, T cells, NK cells*	
MHC-unrestricted CD3(−) NK-LGLs without TCR, which may be positively triggered by circulating antibody against red-cell antigens activating NK-receptors and adhesion molecules [17].	
Antibody cross-linking the TCR of the T-LGLs with the Fc receptor on target cells [19, 20].	
Antibody cross-linking the Fc receptor on the T-LGLs or NK-LGLs (CD16) with any specific ligand for the antibody on the target cells [19, 20].	

**Table 4:** Therapeutic strategies for PRCA [5].

Corticosteroids
Cytotoxic immunosuppressive treatments (cyclophosphamide)
Cyclosporine A
Antithymocyte globulin
Splenectomy
Plasmapheresis
Anti-CD20 monoclonal antibody (rituximab)
Anti-CD52 monoclonal antibody (alemtuzumab)
Intravenous immunoglobulin
Thymectomy
Peptide-based agonists for the erythropoietin receptor

## References

[1] Kaznelson P (1922). Zur entstehung der blutplättchen. *Verhandlungen der Deutschen Gesellschaft für Innere Medizin*.

[2] Maciejewski JP, Tiu RV, Hoffman R, Benz EJ, Shattil SJ (2009). Acquired disorders of red cell and white cell production. *Hematology: Basic Principles and Practice*.

[3] Vlachaki E, Tselios K, Charalambidou S, Ioannidou E, Klonizakis I (2008). Pure red cell aplasia complicating B cell small lymphocytic lymphoma: a case report. *International Journal of Hematology*.

[4] Anastasiadis A, Margaritis D, Kotsianidis I (2009). Pure red cell aplasia as first manifestation of splenic marginal zone lymphoma-successful treatment with rituximab: a case report. *Cases Journal*.

[5] Sawada K, Fujishima N, Hirokawa M (2008). Acquired pure red cell aplasia: updated review of treatment. *British Journal of Haematology*.

[6] Dessypris EN, Lipton JM, Greer JP, Foerster J, Lukens JN, Rogers GM, Paraskevas MD, Glader B (2004). Red cell aplasia. *Wintrobe’s Clinical Hematology*.

[7] Hirokawa M, Sawada KI, Fujishima N (2009). Acquired pure red cell aplasia associated with malignant lymphomas: a nationwide cohort study in Japan for the PRCA Collaborative Study Group. *American Journal of Hematology*.

[8] Sung HJ, Kim SJ, Lee JH (2007). Persistent anemia in a patient with diffuse large B cell lymphoma: pure red cell aplasia associated with latent Epstein-Barr virus infection in bone marrow. *Journal of Korean Medical Science*.

[9] Suzuki T, Kumagai T, Kitano A (2007). Pure red cell aplasia successfully treated with rituximab in a patient with relapsed diffuse large B-cell lymphoma. *Rinsho Ketsueki*.

[10] Castelli R, Vismara A, Pavia G, Dagani R, Porro T (2002). Relapsing pure red cell aplasia associated with B-cell chronic lymphocytic leukemia successfully treated by intravenous immunoglobulin concentrate. *Annali Italiani di Medicina Interna*.

[11] Narra K, Borghaei H, Al-Saleem T, Höglund M, Smith MR (2006). Pure red cell aplasia in B-cell lymphoproliferative disorder treated with rituximab: report of two cases and review of the literature. *Leukemia Research*.

[12] Choi JH, Oh YH, Park IK (2010). A case of pure red cell aplasia associated with angioimmunoblastic T-cell lymphoma. *Cancer Research and Treatment*.

[13] Kuramoto K, Oda K, Katsutani S (1998). Acquired pure red cell aplasia associated with relapsed non-Hodgkin’s lymphoma: a case report-improvement of PRCA after acute hepatitis. *Rinsho Ketsueki*.

[14] Go RS, Li CY, Tefferi A, Phyliky RL (2001). Acquired pure red cell aplasia associated with lymphoproliferative disease of granular T lymphocytes. *Blood*.

[15] Alter R, Joshi SS, Verdirame JD, Weisenburger DD (1990). Pure red cell aplasia associated with B cell lymphoma: demonstration of bone marrow colony inhibition by serum immunoglobulin. *Leukemia Research*.

[16] Fisch P, Handgretinger R, Schaefer HE (2000). Pure red cell aplasia. *British Journal of Haematology*.

[17] Handgretinger R, Geiselhart A, Moris A (1999). Pure red-cell aplasia associated with clonal expansion of granular lymphocytes expressing killer-cell inhibitory receptors. *New England Journal of Medicine*.

[18] Ikeda H, Lethé B, Lehmann F (1997). Characterization of an antigen that is recognized on a melanoma showing partial HLA loss by CTL expressing an NK inhibitory receptor. *Immunity*.

[19] Loughran TP (1993). Clonal diseases of large granular lymphocytes. *Blood*.

[20] Loughran TP, Draves KE, Starkebaum G (1987). Induction of NK activity in large granular lymphocyte leukemia: activation with anti-CD3 monoclonal antibody and interleukin 2. *Blood*.

[21] Maini MK, Casorati G, Dellabona P, Wack A, Beverley PCL (1999). T-cell clonality in immune responses. *Immunology Today*.

[22] Tefferi A, Li CY, Witzig TE, Dhodapkar MV, Okuno SH, Phyliky RL (1994). Chronic natural killer cell lymphocytosis: a descriptive clinical study. *Blood*.

[23] Nash R, McSweeney P, Zambello R, Semenzato G, Loughran TP (1993). Clonal studies of CD3- lymphoproliferative disease of granular lymphocytes. *Blood*.

[24] Heegaard ED, Brown KE (2002). Human parvovirus B19. *Clinical Microbiology Reviews*.

[25] Poplar S, Allford S, Rooney N (2010). Chronic parvovirus B19 infection leading to red cell aplasia following treatment of Hodgkin lymphoma: images in haematology. *British Journal of Haematology*.

[26] Hauswirth AW, Skrabs C, Schützinger C, Gaiger A, Lechner K, Jäger U (2007). Autoimmune hemolytic anemias, Evans’ syndromes, and pure red cell aplasia in non-Hodgkin lymphomas. *Leukemia and Lymphoma*.

[27] Srinivas U, Mahapatra M, Saxena R, Pati HP (2007). Thirty-nine cases of pure red cell aplasia: a single center experience from India. *Hematology*.

[28] Kwee TC, De Klerk JM, Nievelstein RA (2011). Imaging of bone marrow involvement in lymphoma: state of the art and future directions. *TheScientificWorldJournal*.

